# Editorial: The Reasoning Brain: The Interplay between Cognitive Neuroscience and Theories of Reasoning

**DOI:** 10.3389/fnhum.2016.00673

**Published:** 2017-01-05

**Authors:** Vinod Goel, Gorka Navarrete, Ira A. Noveck, Jérôme Prado

**Affiliations:** ^1^Psychology Department, York UniversityToronto, ON, Canada; ^2^Center for Social and Cognitive Neuroscience, School of Psychology, Universidad Adolfo IbáñezSantiago de Chile, Chile; ^3^Institut des Sciences Cognitives Marc Jeannerod, Centre National de la Recherche Scientifique and Université de LyonBron, France

**Keywords:** logic, rationality, inference, neuroimaging, deduction, induction, emotions, brain

The ability to reach logical conclusions on the basis of prior information is central to human cognition. Yet, it is generally agreed that the state of our knowledge regarding the mechanisms underlying logical reasoning remains incomplete and highly fragmented (e.g., Khemlani and Johnson-Laird, 2012). The emergence of functional neuroimaging over the past 20 years—and its ability to examine reasoning at the level of recruitment of cortical systems—provides an additional source of data to, not only better understand reasoning as a phenomenon, but to test different theoretical approaches. This has the potential to both prune the number of theoretical explanations of reasoning, but also to expand the space of possibilities in directions unanticipated by behavioral data. This Research Topic explores the extent to which neuroimaging and brain-lesion studies have informed cognitive theories of reasoning. It includes a selection of 20 empirical and theoretical papers from 69 authors. Below we briefly review these papers by breaking them down into two types of contribution, (i) original research articles, and (ii) review and methodological articles.

## Original research articles

Most contributions are original research articles that further our understanding of the reasoning brain in several important ways. Perhaps the main finding from these studies is that reasoning relies on a heterogeneous cerebral network that is task-dependent, as can be seen from functional neuroimaging, brain-lesion, and behavioral studies. For example, Liang et al. use neuroimaging data to show that different neural systems contribute to semantic bias and conflict detection in the inclusion fallacy task. Smith et al. and Smith et al. further demonstrate that the neural bases of logical syllogisms can be modulated by the emotional context of the task. Pamplona et al. also provide evidence that general intelligence modulates connectivity between brain regions underlying reasoning. Using a behavioral approach, Andrews et al. show that a frontal-based domain-general capacity for relational processing is particularly important for tasks that require planning, whereas Vendetti et al. find hemispheric differences in the encoding of ordered vs. out-of-order premises in relational reasoning tasks. Finally, Ye et al. demonstrate a causal relationship between activity in the temporo-parietal cortex and tasks relying on mental state attribution for moral judgment.

The fact that the brain network for reasoning is heterogeneous, however, does not imply that some regions are not more important than others for reasoning. This is notably the case for the Inferior Parietal Lobule (IPL), which is related to several different aspects of reasoning in perspective taking tasks (Arora et al.), and is consistently found activated in reasoning tasks (Wendelken). The importance of the IPL is also illustrated by Hinton et al. who show that enhanced activity in the parietal cortex may be critical for compensating reasoning deficits in sub-clinically depressed participants.

## Review and methodological articles

Other contributions to the Research Topic are reviews and opinions that speculate on the link between cognitive neuroscience research and theories of reasoning. For example, Oaksford reviews some of the brain imaging research on deductive reasoning and argues that this literature could benefit from adopting the probabilistic and dual-system frameworks of reasoning. Oaksford is notably challenged by Bonatti et al. who argue that neuroscience research has made clear progress within these last 15 years, and does not have much to gain from adopting such frameworks. Other important theoretical contributions are those of Khemlani et al. who illustrate how cognitive neuroscience research can inspire a novel computational theory of how individuals segment perceptual information into representations of events. In a similar vein, Houdé and Borst show how cognitive neuroscience can be used to test an inhibitory-control theory of the reasoning brain, which stresses the importance of inhibiting misleading heuristics when activating logical algorithms.

Six contributions are more methodologically driven and argue for changes in the way cognitive neuroscience research on reasoning is done. Papo argues that the study of reasoning in the brain must rely on the development of a new set of non-standard brain metrics, experimental designs, and analytical tools. Roser et al. propose that a useful way to advance investigations of the reasoning brain would be to integrate several neuroscience methods within a single study. Heit argues that a greater use of “forward inference” in interpreting cognitive neuroscience data may settle disputes between competing cognitive theories. Rotello and Heit caution how misinterpretation of behavioral data could lead to the wrong conclusions at the neuropsychological level. Cummins emphasizes the importance of taking into account how knowledge is activated and weighted in decision processes in the modeling of human causal inference. Finally, Beatty and Vartanian point out that cognitive research on reasoning might also have practical implications. For example, the fact that reasoning is intrinsically linked to working-memory suggests that working memory training could lead to important improvements in reasoning.

Have neuroimaging and brain-lesion studies enhanced our understanding of human reasoning? The main contribution of the augmentation of behavioral data with neuropsychological data has been to question unitary accounts and advocate for the engagement of multiple cognitive systems in reasoning. That is, rather than simply pruning the space of possibilities provided by mental models, mental logic, dual mechanism, and probabilistic account theories, the effect of the neuropsychological data has been to expand the search space in ways not foreseen by behavioral data. This does not make the contribution any less valuable. It identifies challenges, issues, and directions for future research. We hope that readers find this Research Topic informative, thought provoking, and helpful in moving forward the understanding of the cognitive and neural basis logical reasoning.

## Author contributions

All authors listed, have made substantial, direct and intellectual contribution to the work, and approved it for publication.

### Conflict of interest statement

The authors declare that the research was conducted in the absence of any commercial or financial relationships that could be construed as a potential conflict of interest.
